# Disruption of blood pressure circadian rhythm in children with obstructive sleep apnea—is it a pathway leading to cardiovascular morbidities?

**DOI:** 10.1093/sleep/zsad284

**Published:** 2023-12-13

**Authors:** Kate Ching-Ching Chan, Albert M artin Li

**Affiliations:** Department of Paediatrics, Faculty of Medicine, The Chinese University of Hong Kong, Hong Kong SAR, China; Laboratory for Paediatric Respiratory Research, Li Ka Shing Institute of Health Sciences, Faculty of Medicine, The Chinese University of Hong Kong, Hong Kong SAR, China; Hong Kong Hub of Paediatric Excellence, The Chinese University of Hong Kong, Hong Kong SAR, China; Department of Paediatrics, Faculty of Medicine, The Chinese University of Hong Kong, Hong Kong SAR, China; Laboratory for Paediatric Respiratory Research, Li Ka Shing Institute of Health Sciences, Faculty of Medicine, The Chinese University of Hong Kong, Hong Kong SAR, China; Hong Kong Hub of Paediatric Excellence, The Chinese University of Hong Kong, Hong Kong SAR, China

Obstructive sleep apnea (OSA) is a recognized risk factor for elevated blood pressure (BP) and hypertension in both children and adults [1]. However, how OSA affects the BP circadian rhythm is a less studied area. Most of the body’s physiologic mechanisms, including BP, follow a circadian pattern through a complex and mutual interplay between the intrinsic biologic clock and the autonomic, hormonal, environmental, and behavioral factors [2]. Disruption of the BP circadian patterns, for instance, excessive morning BP surge [3, 4] and reduction in nocturnal BP dipping [5, 6], has been associated with adverse cardiovascular and cerebrovascular outcomes. In adults, OSA is associated with reduced nocturnal BP dipping. Compared to controls, children with OSA are at increased risk of elevated BP, with the effects more pronounced during the night [1]. However, whether reduced nocturnal dipping occurs in children with OSA remains controversial. Reduced nocturnal dipping has been documented in some studies [7, 8] but not in others [9–11]. Research studying the BP circadian patterns over 24 hours in childhood OSA is also limited.

Khan et al. set out to investigate the 24-hour circadian BP rhythms in 102 children with OSA (52 and 50 with mild and moderate-to-severe OSA, respectively) compared to 117 age- and gender-matched healthy non-snoring controls through ambulatory BP monitoring [12]. OSA was defined by the obstructive apnea–hypopnea index (oAHI) derived from overnight polysomnography and was categorized as mild if oAHI was ≥1 and <5 and moderate-to-severe if oAHI ≥ 5. BP circadian patterns included the times of BP peaks and troughs and the time arrived at peak BP velocity (TAPV). The Super Imposition by Translation And Rotation model was applied to the 24-hour BP data to identify OSA-related changes in BP patterns and rhythmicity. The 24-hour BP data were synchronized so that time zero was designated as a time of sleep onset, which was confirmed by actigraphy, aiming to account for the variations in the timing of sleep and wake states among the children. It was found that children with moderate-to-severe OSA (MS-OSA) demonstrated earlier TAPV, BP peaks, and nadirs than controls. TAPV for diastolic BP (DBP) in the morning was 51 minutes earlier in children with MS-OSA than controls, and TAPV for systolic BP (SBP) and DBP in the evening was 95 and 28 minutes earlier in MS-OSA than controls, respectively. Similarly, children with MS-OSA showed earlier SBP and DBP velocity nadirs than controls. Compared to controls, those with MS-OSA also had higher BP and were more inclined to nocturnal BP non-dipping. Interestingly, distinct periods of peaks and troughs of SBP and DBP were observed in children with MS-OSA compared to controls: SBP was elevated in the evening, while DBP was elevated in the morning and early afternoon in children with MS-OSA.

BP control has been more meaningfully redefined relative to specific times of the day, based on the evidence that disruption of circadian BP pattern and elevated nocturnal BP are more strongly associated with cardiovascular risk than daytime BP parameters alone [4, 5]. Therefore, preserving circadian BP regulation (including 24-hour control, adequate circadian rhythm, and appropriate BP variability), and not only the BP levels, is important from the perspective of cardiovascular health. The circadian disturbances in BP rhythms observed in children with MS-OSA may reflect BP dysregulation and a pathway leading to future cardiovascular adverse events. However, the clinical relevance of the different timing in TAPV, BP peaks and troughs observed in children with MS-OSA compared to controls still requires further investigation.

In recent years, studies have looked at the circadian disturbance in individuals with OSA based on the crosstalk between hypoxia, arousals, and the circadian clock [13, 14]. It has been proposed that exposure to oxygen desaturations and/or sleep fragmentation during critical periods of sleep may result in circadian disruption or misalignment [13–16]. Studies have demonstrated the alteration of clock gene expression profiles in adult patients with OSA compared to controls [16–19]. Dysregulation of circadian rhythms may also be involved in the pathogenesis of OSA-related comorbidities, such as systemic inflammation, hormonal homeostasis, and BP regulation [14]. However, it is known that BP is regulated through various pathways, including the autonomic nervous system, renal and hormonal axis. Although the study demonstrated potential differences in the BP circadian rhythms, the mechanisms leading to such differences require further investigation. It remains to be explored whether the changes in BP circadian rhythms are caused by the disturbance in the circadian regulation or secondary to other pathways such as autonomic dysfunction or hormonal dysregulation. Identifying the underlying pathophysiology of disturbed BP circadian patterns may pave the way to explore new interventional strategies [2]. Moreover, in contrast to the findings reported by Khan et al., another study found that the circadian rhythm of BP peaked later in adults with OSA compared to controls, with a trend toward a delayed cortisol rhythm in the OSA group [20]. More studies are needed to optimize the methodology in investigating circadian BP regulation in individuals with OSA and confirm the differences or disturbances identified. In future studies, it would also be invaluable to incorporate measures to investigate circadian biology in children with OSA, such as actigraphy and biomarkers like melatonin and cortisol, as well as circadian gene expression profiles.

In conclusion, Khan et al. identified the differences in the circadian BP patterns in children with OSA compared to healthy controls. The findings provide an essential puzzle piece in our understanding of the cardiovascular effects of OSA in children. On the way forward, future research is needed to ascertain the observation and elucidate the natural history and clinical implications of circadian BP rhythm disruptions in children with OSA.
